# Fruit quality and shelf-life of Sardinian tomato (*Solanum lycopersicum* L.) landraces

**DOI:** 10.1371/journal.pone.0290166

**Published:** 2023-12-08

**Authors:** Chiara M. Posadinu, Monica Rodriguez, Paola Conte, Antonio Piga, Giovanna Attene

**Affiliations:** 1 Department of Agriculture, University of Sassari, Sassari, Italy; 2 Centro Interdipartimentale per la Conservazione e Valorizzazione della Biodiversità Vegetale, University of Sassari, Alghero, Italy; University of Verona: Universita degli Studi di Verona, ITALY

## Abstract

The conservation and characterization of landraces have key roles in the safeguarding and valorization of agrobiodiversity. Indeed, these plant genetic resources represent an important crop heritage with quality and sensory characteristics that can be of great use to consumers and industry. In addition, the preservation of genetic resources from the risk of progressive genetic erosion, and the enhancement of their potential can contribute to food security and improve the nutritional value of food. Accordingly, this study aimed to investigate a collection of Sardinian tomato landraces for parameters that have determinant roles in evaluating their responses to conservation, and therefore to consumer acceptance. Six Sardinian landraces and two commercial varieties were cultivated in a two-years off-season trial, harvested at two different maturity stages (turning, red-ripe) and characterized using 14 fruit-related quality parameters that define the marketability, nutritional value, and flavor of the fruit. Data were collected at intervals of 10 days, starting from the harvest date and over 30 days of storage under refrigeration. The simultaneous analysis of all the qualitative characteristics for the different genotypes allowed to clearly differentiate the local varieties from the commercial varieties and a few landraces emerged for their satisfactory performances, e.g. “Tamatta kaki” ad "Tamatta groga de appiccai". In particular, the “Tamatta groga de appiccai” showed satisfactory lycopene content at marketable stages (average 5.65 mg 100g^-1^ FF), a peculiar orange-pink color with the highest hue angle values (range: H°_T0_ = 72.55—H°_T30_ = 48.26), and the highest firmness among the landraces of the red-ripe group (range: Ep_T0_ = 1.64—Ep_T30_ = 0.54 N mm^-1^). These results highlight the potential of some of the Sardinian tomato landraces for developing new varieties or promoting their direct valorization in local markets and could considerably increase the effectiveness and efficiency of agrobiodiversity conservation strategies.

## Introduction

In recent years, farmers have significantly increased their use of modern crop cultivars, commercial varieties, and hybrids due to market trends and modern vegetable growing systems [1]. For a long time, the agri-food industries and breeding programs favored varieties with high yield, good resistance to various diseases, uniformity of the final product, and long shelf-life [1–4]. Currently, however, the demand of consumers is directed toward good quality products with high nutritional and sensory values, with local varieties consistently under increasing attention [1, 5–7]. The quality of horticultural products mainly depends on genotypic, agri-environmental, and harvesting and postharvest factors [8–12], although these are also influenced by socioeconomic and marketing forces that can condition consumer perception of a product [13, 14]. To meet consumer demand, the activities of breeding programs have recently started to look for improvements to qualitative characteristics, in both sensorial and commercial terms [2, 15–18].

In this context, the potential value of plant genetic resources such as locally adapted varieties (i.e., ‘landraces’) has been recognized, in that these can include characteristics that can be useful to respond to the changing demands of society, in terms of food production and consumption [19–22]. The interest in tomato (*Solanum lycopersicum* L.) derives from its importance as one of the main horticultural crops grown and consumed around the world [23]. The high consumption of tomatoes worldwide is justified by the appreciation of this vegetable by consumers, especially for its high nutritional value, and also for the versatility of its use, both fresh and processed. Moreover, Italy is the highest producer of tomatoes in Europe which currently occupy a total area of over 772 ha in both open fields and greenhouses cultivations with an overall production of over 6 million tons [23, 24]. Indeed, Italy, a recognized secondary center of diversification [25–28], remains one of the richest countries in terms of ancient tomato varieties that are still present and used by local farmers for mainly private consumption, many of which have been specifically recognized as quality products [28–31]. Cultiovation of tomato has a long tradition also in Sardinia, being tomato the most common vegetable, together with the artichoke and the potato. In 2022, the cultivated area was about 613 hectares considering both open-field cultivations conducted during the spring-summer season, and greenhouse farming systems of the autumn-winter and spring-summer seasons. The total Sardinian production in 2022 did not reach 82000 tons [23, 24].

Numerous studies have been conducted with the aim to define the variability of tomato landraces, also in comparison with those of modern commercial varieties [28, 31–35]. These studies represent an example of the importance of the characterization of local varieties, which can provide useful information on a series of parameters related to the physicochemical, nutraceutical, and color of the fruit.

Indeed, the scientific community is increasingly engaged in research for innovative strategies to improve crop quality [15, 16, 36] and commercial characteristics [37, 38] to meet consumer demand. Different quality attributes have been defined, in terms of sensorial quality, appearance, and shelf-life of the products [39–41]. Among all, biochemical components (e.g., antioxidants, sugars, acids, and volatile compounds) [5, 18, 42, 43] and the external appearance of the product (e.g., color, shape, size, defects, and firmness) [42, 44, 45] are the most important attributes of fruit quality. They are also critical to determine, as they change during fruit ripening and storage [42, 46–49] and they define the nutritional value and the overall flavor of tomato fruit [5, 43, 50–53], thus influencing its commercial quality and value [6, 12]. The shelf-life of products is also a key characteristic that is associated with the postharvest quality of food [17]. The shelf-life of horticultural products, such as tomato fruit, can be shortened by different factors, such as maturity stage at harvest, postharvest conditions, and pathogen diseases [17, 54, 55]. Also, preharvest factors can have effects on tomato fruit mass, firmness, and composition, such as the environment (e.g., light intensity, temperature, air humidity) and the horticultural management (e.g., water, fertilization, grafting, pruning) [18, 35].

This research is part of a wider project aimed at comparing the response of different local tomato varieties (landraces and commercial varieties) grown in greenhouse conditions during the autumn/winter season under modern horticultural techniques. Cultivating tomato landraces in a season when they are generally not cultivated and in conditions never studied before for this type of variety was one of the project goals.

Accordingly, in the present study, the effects of storage on tomato landraces from Sardinia (Italy) were compared to those on commercial varieties (six local Sardinian varieties; two modern cultivars), in terms of the changes in their quality characteristics over 30 days of storage under refrigeration. These varieties were characterized using 14 fruit-related quality parameters that were defined by physicochemical and texture analyses, performed at 10-day intervals starting from the harvest date. The external appearance of the fruit was evaluated, including color, size, defects, and firmness, as these are the main factors considered by consumers. In addition, as the sensory characteristics and nutritional value of these fruit are becoming progressively important, these were also determined, including the contents of antioxidants, sugars, and acids [5, 18, 40, 41, 56].

The goals of the present study were: (a) to compare landraces and commercial varieties for the main characteristics directly related to the intrinsic quality of the fruit and their preservation, as components that are closely related to the commercial and nutritional value; and (b) to look for the tomato landraces that show valuable post-harvest qualities after refrigerated storage conditions. The results can be of use for direct valorization of landraces in local markets and production and/or in future breeding programs.

## Materials and methods

### Plant materials

The plant materials were selected from a collection of Sardinian tomato landraces, mainly collected during 2006 and 2007 which were evaluated during the spring-summer season in two different trials, one conducted in 2012 in open field and the other in 2013 in greenhouse [57]. The materials selected for the present study were further evaluated in greenhouse during a two-years trial, referred to in this study as Experiments #1 and #2 (Exp#1 and Exp#2, respectively). The collection of Sardinian tomato landraces is owned by the “Centro Interdipartimentale per la Conservazione e Valorizzazione della Biodiversità Vegetale” (CBV) of the University of Sassari (Italy), which kindly granted permission to use the plants evaluated in the present study.

The materials evaluated during Exp#1 included one Italian vintage variety, three commercial varieties, and 12 landraces with fruit of variable shape and size. Based on the results obtained from Exp#1, six Sardinian local varieties, one Italian vintage variety, and three commercial varieties were included in Exp#2.

Shelf-life trials were set up for a subset of the tested varieties: four Sardinian tomato landraces (P01, P05, P16, P46) and two commercial varieties (C1, C3) were tested in the Exp#1, for a total of six different accessions; two additional landraces (P04, P44) were included in the Exp#2, thus the samples under evaluation were six landraces and two commercial varieties (S1 Table). Overall, therefore, during the two-years trial we evaluated the fruit quality and shelf-life of eight different accessions. The accessions were selected among four different types of cultivars (e.g., beefsteak, oxheart, plum, and round) commonly present in Sardinia, all cultivated for fresh consumption and having different fruit characteristics (S1 Table). The commercial varieties were chosen among those cultivated and marketed during the autumn/winter season by the farmer partner of the project. These are representative of the different cultivar groups evaluated in the present research.

### Field trials

A randomized complete block design with three replicates was adopted for the two trials, which were run under greenhouse conditions during the autumn-winter season of 2017 to 2018 (Exp#1) and 2018 to 2019 (Exp#2). The trials were conducted on the specialized horticultural farm “Società Agricola F.lli Scintu” (36°55’45.36” N, 8°37’39.88” E) in Oristano, Sardinia (Italy). The company is a partner of a bigger project of which this study is part. The field was characterized by 5 mulched rows (3 as experimental trials and 2 as borders), with 1.70 m of distance between the rows and plants spaced 0.40 m apart in-the-row. Plants of commercial tomato varieties were transplanted all around the field as borders of the trials. The same modern horticultural cultivation techniques and agronomic practices of the farm, which are different from the traditional practices applied for tomato landraces cultivation, have been adopted: all plants were staked by cords and clips on two stems, constantly pruned during the growth and, when the plants reached the height of about 1.8 m, the apex was trimmed. During the early stages of plants development, 500 mL of water per plant were administered in 9 minutes, when necessary, by drip irrigation. From the setting of the second flower truss until reaching about 1.20 m in height, the plants were fertigated daily by administering 1 l of solution per plant in 12 minutes. After the trimming of the apex of the plants, however, the irrigation was carried out only occasionally. Moreover, the cultivation was carried out by adopting integrated pest management.

### Storage conditions and shelf-life experiment

The fruits were harvested at two different stages of ripeness following three different criteria: the tomato color classification according to the US Department of Agriculture (USDA) standard chart [58], the commercial category of the varieties and the local marketing-related harvesting standards. Three Sardinian landraces (P01, P05, P44) and one commercial variety (C3) were harvested at the ‘red-ripe stage’, and three further Sardinian landraces (P04, P16, P46) and another commercial variety (C1) were harvested at the ‘turning stage’ (S1 Table). Whereby, the varieties that belonged to the plum, mini plum, and round tomato commercial categories were harvested at the “red-ripe stage”, while the varieties that belonged to the beefsteak and oxheart tomato commercial categories were harvested at the “turning stage” (S1 Table). The fruit were randomly collected above the third flower truss from all plants of each variety present in the trial.

Following harvesting, the fruit were selected for uniformity in size and color, and absence of visual defects and fungal infection, and then transferred to the laboratory at the University of Sassari for analysis. The fruit were stored for 30 days at 13°C and 90% relative humidity to reduce tomato decay and maintain tomato fruit quality while avoiding compromising tomato flavor due to chilling injury [59–62]. The analyses were performed at intervals of 10 days, mostly according to the procedures described by D’Aquino et al. [63]. A total of four inspection times were included, as date of harvest (T0) and 10, 20 and 30 days postharvest (T10, T20, T30). Fourteen physicochemical and texture parameters were used to characterize each variety at each storage time (S2 Table). A total of 12 fruit for each variety harvested at the turning stage and 24 fruit for each variety harvested at the red-ripe stage were stored (3 and 6 fruit for each storage time, respectively), with the exception of the commercial variety C3, for which 30 fruit were included for each storage time, due to the small size of these fruit.

#### Color assessment

For each sample, color assessment was carried out in triplicate on five different points along the fruit equator, using a colorimeter (CR 300; Konica Minolta Sensing, Osaka, Japan). The color assessment was repeated at each storage time on the same fruit used to quantify the weight loss for each variety. The CIE-Lab values were recorded, where: L* quantifies lightness, ranging from 0 (black) to 100 (white); a* quantifies greenness (negative values) to redness (positive values); and b* quantifies blueness (negative values) and yellowness (positive values). Before measurements, the colorimeter was calibrated with a standard white plate. The hue angle (*H°*) was calculated according to Eq (1) [64].


H°=tan−1[b*/a*] (1)

The hue angle is an indicator of the chromatic nature of the color and defines the color traditionally as reddish, yellowish, and greenish [65, 66]. The hue angle is expressed in degrees, and ranges from 0° to 360° (0°, 90°, 180°, 270° as pure red, yellow, green, blue, respectively).

#### Chemical analyses and lycopene determination

All of the chemical analyses were performed in triplicate using the filtered juice of the fruit obtained by homogenization of 100 g tomato fruit in a domestic blender. The juice was centrifuged at 13,000× *g* for 15 min, and the supernatant was filtered through 0.45-μm cellulose acetate filters.

The pH was measured using a pH meter (Orion 420A; Thermo Fisher Scientific, Waltham, USA), the total soluble solids (*TSS*) were measured with a digital refractometer (PR-101 Palette series; Atago Co., Ltd, Japan) and are expressed as °Brix. The titratable acidity (*TA*) is given as g L^-1^ citric acid in the juice after titration of 5 g juice with 0.1 N NaOH, to an endpoint of pH 8.2.

Lycopene content was determined as described by Kopec et al. [67]. Briefly, 50 mL hexane/acetone/ethanol (2:1:1) was added to 5 g homogenized fresh tomato sample. The solution was agitated in a glass vial wrapped with aluminum foil, to exclude light. After 60 min, the nonpolar layer that contained lycopene was measured at 470 nm using a UV–Vis spectrophotometer (8453; Hewlett–Packard, Palo Alto, CA, USA). The calibration curve was obtained using different concentrations of the lycopene standard and was used to determine the total lycopene content (mg 100 g^-1^ FF).

#### Weight loss

Before storage, five additional fruit for each variety were selected to determine the weight loss (*WL*). These fruit were individually weighed at harvest and at each of the four storage times. The weight loss was calculated as percentage reduction of the initial weight.

#### Texture assessments

Texture analysis was carried out for each fruit using a texture analyzer (TA-XT2 Plus; Stable Micro Systems, Surrey, UK), and the texture parameters obtained were entered automatically into the Texture Exponent 32 software (Stable Microsystems, Surrey, UK). A penetration test was performed using three fruit for each variety and storage time, to monitor changes in the samples during storage. For the puncture test, the fruit were placed on a platform and punctured in the lateral face by a needle probe of 2-mm diameter. A final depth of 1.5 cm at a speed of 1.7 mm s^-1^ was reached, and the force applied was recorded. Four parameters were registered: the force required to puncture the tomato skin (*Fp*, [N]), which represented the skin rupture; the probe position at *Fp* (*Dp*, [mm]) which determined the fruit deformation before skin rupture; the mechanical work necessary to reach the breaking point (*Wp*, [N mm]), as the area under the curve up to the skin rupture point; and the firmness (*Ep*, [N mm^-1^]), which represented the slope of the first part of the curve, as defined by *Fp/Dp*.

#### Visual quality

On the same five additional fruit selected to determine the weight loss for each variety, the overall appearance (visual quality; *VQ*) was also evaluated. As indicated by D’Aquino et al. [63], the overall appearance was determined at each storage time based on the perception of visual defects (e.g., shriveling, bruising, pitting) and loss of firmness after slight pressure exerted by the fingers. The overall visual quality was determined using a nine-point hedonic scale, where: 1 = very poor (severe presence of pitting, general decay [>50%] and total loss of firmness); 3 = poor (moderate presence of visual defects, 6% to 50%); 5 = good, limit of marketability (1% to 5% defective, slight loss of firmness and mild presence of shriveling); 7 = very good (no pitting and decay on the fruit surface and very slight loss of firmness); 9 = excellent (0% of damage and fruit very firm) (S1 Fig) [63]. The end of the shelf-life was defined as when the appearance of the majority of the fruit for each variety was below the limit of marketability.

### Statistical analysis

All of the statistical analyses were performed independently for each group (i.e., “red-ripe stage” and “turning stage”) using the JMP version 7 software (SAS Institute, Inc.). The data collected at harvest were analyzed as a first step using a two-way ANOVA to check for possible interactions among varieties and years of cultivation. In a second step, one-way analysis of variance (ANOVA) was used to determine the significant differences between the varieties within storage time, the varieties were selected as the factor. To determine the significant changes during conservation, the storage time was selected as the factor. The means were separated according to Tukey honest significant differences at the p <0.05 level. The Figures were created using MS-Excel 2016.

Multivariate analysis was used to obtain an overview of the whole data variability. Principal component analysis (PCA) was performed on the mean data matrix on 14 parameters and 12 observations (three varieties × four storage times) for Exp#1, and 16 observations (four varieties × four storage times) for Exp#2. Only the principal components (PCs) with eigenvalues ≥1 were retained for data discussion [68], and the correlations between each parameter and the PCs were calculated and loadings >|0.6| were considered for further discussion. The results of the PCA are shown as biplots of scores (varieties × storage time) and loadings (variables), and were drawn using RStudio 1.1.423 (RStudio, Inc. 2016, Boston, MA, USA).

## Results

### Comparison between trials and materials selection

Previous results from Rodriguez et al. [57], were used to choose the materials to test in the first greenhouse trial (Exp#1); a group of 12 local varieties was chosen to be representative of the morpho-phenotypic and molecular variability of the collection; all of the chosen varieties have an indeterminate growth habit. The results obtained from the Exp#1 were used to determine which varieties to include in the Exp#2: varieties with an average fruit weight (FWG) above 300 g and usually late ripening (S3 Table) were excluded, also based on the poor performance seen at the modern horticultural cultivation conditions set for the experiment. For the shelf-life trials, a final subset of six landraces were selected to be compared to two commercial varieties commonly cultivated and marketed by the farmer partner of the project.

### Variance of tomato traits at harvest

The two-way ANOVA conducted on the shelf-life traits at harvest (only varieties shared between experiments were included) has allowed to detect some main features. A significant Variety effect and not significant Year and Year x Variety effects were observed for *Ep* (both at the turning and red-ripe stages) and *TA* (at the turning stage), indicating that the genetic effect for these traits was substantial and consistent across years (Table 1). Moreover, the effect Year x Variety interaction was not significant for pH (at both turning and red-ripe stages) and for *H°* (at the turning stage) thus indicating that changes in these traits were consistent across years and genotypes (Table 1). The traits with which Variety x Year interaction effects were found significant were *TSS* and *LYC* at both stages and *H°* and *TA* at the red-ripe stage, indicating a more complex pattern of variance across years and genotypes (Table 1). In particular, interaction of the crossover type was observed for *TSS* and *LYC* at the turning stage and for *H°* and *TA* at the red-ripe stage (S2 Fig).

**Table 1:** ANOVA showing year, variety and year x variety interaction effect on hue angle (H°), firmness (Ep), total soluble solids (TSS), tritatable acidity (TA), and lycopene content (Lyc) evaluated in the two-years trial at harvest. Year, genotype and the interaction year x genotype have been considered as effects of the model.

		H°			Ep (N/mm)				pH		TSS (°Brix)			TA (g CA L^-1^ juice)			Lyc (mg 100 g^-1^ FF)		
Effect	DF	SS	F	P	SS	F	P	SS	F	P	SS	F	P	SS	F	P	SS	F	P
										Turning									
Year	1	426.95	22.56	****	0.00	0.00	n.s.	0.01	5.32	*	0.01	0.15	n.s.	0.07	0.22	n.s.	7.30	22.21	***
Variety	2	621.75	16.43	****	2.25	67.29	****	0.05	11.91	**	0.39	3.36	n.s.	3.86	6.26	*	2.28	3.46	n.s.
Year*Variety	2	69.40	1.83	n.s.	0.00	0.00	n.s.	0.00	0.96	n.s.	0.97	8.43	**	1.93	3.14	n.s.	12.34	18.78	***
** **	** **	** **	** **	** **	** **	** **	** **	** **	** **	**Red-ripe**	** **	** **	** **	** **	** **	** **	** **	** **	** **
Year	1	99.18	26.69	****	0.02	2.99	n.s.	0.01	8.53	*	0.85	21.42	***	0.02	0.15	n.s.	0.06	0.05	n.s.
Variety	2	170.08	22.89	****	0.44	26.94	****	0.01	8.55	**	31.45	398.70	****	1.25	4.78	*	171.63	69.28	****
Year*Variety	2	166.43	22.40	****	0.01	0.45	n.s.	0.00	1.50	n.s.	0.89	11.32	**	3.90	14.95	***	37.94	15.32	***

H, Hue angle; Ep, Firmness; TSS, total soluble solids; TA, titratable acidity; CA, citric acid; LYC, lycopene; FF, fresh fruit

DF, degrees of freedom; SS, sum of squares; F, F ratio; P, p-value

n.s. not significant

* P < 0.05

** P < 0.01

*** P < 0.001

**** P < 0.0001

### Changes in fruit color and lycopene content

For *H°*, as the hue indicator that defines the colors as reddish, yellowish, and greenish, in the turning stage fruit, this decreased during the storage for both experiments. There were significant differences among the sampling times for almost all of the varieties, with an increase of the redness of the fruit (*H°* = 0° indicates completely red) (Table 2). For the turning stage, the commercial variety (C1), which in Exp#1 showed the highest *H°* at harvest and T10 (70.90 and 51.54 respectively), experienced the highest degree of *H°* decrease (30.2) compared to landraces (16.9 for P46 and 13.6 for P16). Similarly, in Exp#2 the commercial variety showed the highest *H°* values, this time together with landrace P04, and both showed the highest *H°* decrease across the storage times (37.7 and 33.4, respectively). Significant differences among varieties were registered only at harvest, when landrace P46 was more reddish than C1 and P04 (Table 2).

**Table 2:** Color parameter and lycopene contents for the turning stage and red-ripe groups in both experiments.

Days post-	Variety	Turning stage				Days post-	Variety	Red-ripe stage			
harvest	code	Exp#1 (2017–18)		Exp#2 (2018–19)		harvest	code	Exp#1 (2017–18)		Exp#2 (2018–19)	
		H°	LYC	H°	LYC			H°	LYC	H°	LYC
			(mg 100 g-1 FF)		(mg 100 g-1 FF)				(mg 100 g-1 FF)		(mg 100 g-1 FF)
T0	P04	ni	ni	78.72 ± 3.06aA	2.94 ± 0.32aB	T0	P01	40.59 ± 1.84aA	6.39±1.51bA	48.34 ± 2.70bA	4.48±0.53bB
	P16	58.04 ± 1.69bA	4.28 ± 1.19abB	70.12 ± 6.46abA	2.99 ± 0.28aC		P05	42.85 ± 1.33aA	8.11±1.44abA	46.89 ± 0.69bA	5.68±1.17bA
	P46	59.16 ± 3.80bA	5.48 ± 0.60aB	66.16 ± 5.40bA	2.18 ± 0.01bB		P44	ni	ni	72.55 ± 6.62aA	4.67±1.49bA
	C1	70.90 ± 1.88aA	2.62 ± 0.12bC	75.68 ± 4.42aA	3.38 ± 0.32aB		C3	41.47 ± 2.05aA	10.60±0.75aA	39.64 ± 2.02cA	14.58±0.95aA
T10	P04	ni	ni	50.26 ± 1.72aB	5.88 ± 0.49aA	T10	P01	38.61 ± 1.19bAB	5.33±0.47cA	41.56 ± 0.77bcB	5.45±0.50bB
	P16	48.08 ± 3.34abA	6.69 ± 0.55bAB	49.37 ± 1.36aB	5.34 ± 0.53aB		P05	41.56 ± 1.00aA	8.26±1.67bA	45.78 ± 3.14bA	6.50±2.20bA
	P46	45.40 ± 1.78bB	9.06 ± 0.55aA	44.95 ± 1.26aB	6.33 ± 1.97aA		P44	ni	ni	53.51 ± 6.34aB	5.80±1.10bA
	C1	51.54 ± 2.34aB	3.40 ± 0.68cC	46.20 ± 5.88aB	7.07 ± 0.40aA		C3	40.33 ± 2.08abA	13.13±0.70aA	40.35 ± 1.99cA	11.22±2.59aA
T20	P04	ni	ni	41.78 ± 2.58bC	6.31 ± 0.57bA	T20	P01	37.82 ± 1.43bB	5.18±0.56bA	41.81 ± 0.93bcB	8.17±0.31bA
	P16	44.41 ± 1.83aB	9.05 ± 0.71aA	47.05 ± 1.54aB	6.32 ± 0.62bAB		P05	40.44 ± 1.71aB	6.63±0.91bA	44.73 ± 2.14bA	6.37±0.89bA
	P46	42.94 ± 0.62aB	8.59 ± 0.64aA	42.43 ± 1.64bB	6.91 ± 0.64abA		P44	ni	ni	52.38 ± 3.88aB	6.48±0.82bA
	C1	44.26 ± 3.08aC	5.03 ± 0.93bB	43.68 ± 2.74abB	9.27 ± 1.79aA		C3	39.78 ± 1.47abAB	13.43±1.43aA	39.37 ± 0.45cA	14.70±1.68aA
T30	P04	ni	ni	41.06 ± 2.53bC	6.00 ± 1.19bA	T30	P01	38.2 ± 1.27aAB	6.16±1.06cA	41.99 ± 0.73bB	7.45±1.06abA
	P16	44.45 ± 1.47aB	7.86 ± 2.27aA	47.15 ± 2.11aB	7.56 ± 1.26abA		P05	39.33 ± 1.13aB	9.87±0.98bA	43.63 ± 0.64bA	6.03±1.79bA
	P46	42.25 ± 0.96bB	8.03 ± 0.30aA	42.68 ± 0.93abB	6.28 ± 0.45bA		P44	ni	ni	48.26 ± 3.79aB	6.52±2.33abA
	C1	40.67 ± 0.47bC	7.97 ± 0.24aA	42.26 ± 4.18abB	9.88 ± 1.43aA		C3	38.58 ± 1.21aB	12.91±1.41aA	39.13 ± 0.49cA	10.73±1.20aA

Data are means ±standard deviation (five independent replicates). Means followed by different letters indicate significant differences among the varieties within each storage time (lowercase letters) and among the storage times within variety (uppercase letters) (p <0.05; Tukey-Kramer’s tests).

H°, Hue angle; LYC, lycopene; FF, fresh fruit; ni, not included in experiment. For varieties codes, see S1 Table. For ANOVA details, see S4 and S5 Tables.

The lycopene content changed during storage, with significant differences among the storage times and genotypes for both years (Table 2). In more detail, Exp#1 showed that landrace P46 had the highest lycopene content at harvest (*LYC* = 5.48 mg 100 g^-1^ fresh fruit [FF]), while the commercial variety (C1) had the lowest both at harvest (*LYC* = 2.62 mg 100 g^-1^ FF) and at T10 (*LYC* = 3.40 mg 100 g^-1^ FF) and at T20 (*LYC* = 5.03 mg 100 g^-1^ FF; Table 3). On the contrary, in Exp#2, landrace P46 had the lowest lycopene content at harvest (*LYC* = 2.18 mg 100 g^-1^ FF), followed by the two other landraces (P04, P16) and C1 (Table 2).

**Table 3:** Chemical parameters for the turning stage fruit for both experiments.

Days post-	Variety	Exp#1 (2017–18)			Exp#2 (2018–19)		
harvest	code	pH	TSS	TA	pH	TSS	TA
			(°Brix)	(g CA L^-1^ juice)		(°Brix)	(g CA L^-1^ juice)
T0	P04	ni	ni	ni	4.32 ± 0.03aA	3.80 ± 0.00bB	2.77 ± 0.51cB
	P16	4.23 ± 0.04abA	4.23 ± 0.45aA	4.50 ± 1.15aA	4.28 ± 0.02abA	3.90 ± 0.17bA	4.00 ± 0.40bAB
	P46	4.31 ± 0.08aB	4.13 ± 0.25aA	3.97 ± 0.31aA	4.32 ± 0.05aA	3.90 ± 0.17bA	3.30 ± 0.36bcA
	C1	4.13 ± 0.06bA	4.00 ± 0.10aB	4.37 ± 0.21aA	4.22 ± 0.01bB	4.70 ± 0.10aA	5.17 ± 0.31aA
T10	P04	ni	ni	ni	4.22 ± 0.05bAB	3.73 ± 0.38bB	3.40 ± 0.66abAB
	P16	4.32 ± 0.07aA	3.67 ± 0.57aA	4.40 ± 0.40abA	4.27 ± 0.01abA	3.60 ± 0.20bA	4.40 ± 0.36aA
	P46	4.31 ± 0.01abB	4.07 ± 0.51aA	3.73 ± 0.23bAB	4.33 ± 0.03aA	3.40 ± 0.50bA	2.80 ± 0.44bA
	C1	4.21 ± 0.01bA	4.20 ± 0.17aB	4.77 ± 0.15aA	4.25 ± 0.05abB	4.77 ± 0.15aA	4.03 ± 0.15aB
T20	P04	ni	ni	ni	4.15 ± 0.02bB	5.15 ± 0.21aA	4.17 ± 0.23aA
	P16	4.34 ± 0.03bA	3.43 ± 0.12bA	4.13 ± 0.38aA	4.47 ± 0.1aA	3.47 ± 0.12bA	3.10 ± 0.44bAB
	P46	4.45 ± 0.01aA	3.60 ± 0.260bA	3.13 ± 0.06bBC	4.29 ± 0.07bA	3.73 ± 0.35bA	2.97 ± 0.32bA
	C1	4.17 ± 0.02cA	4.30 ± 0.00aB	4.80 ± 0.36aA	4.28 ± 0.05bB	4.87 ± 0.38aA	4.30 ± 0.44aAB
T30	P04	ni	ni	ni	4.33 ± 0.07aA	4.07 ± 0.40abB	3.30 ± 0.40abAB
	P16	4.31 ± 0.3aA	3.77 ± 0.68bA	3.93 ± 1.12abA	4.53 ± 0.25aA	3.77 ± 0.23bcA	2.73 ± 0.74bB
	P46	4.44 ± 0.01aA	3.70 ± 0.20bA	3.10 ± 0.26bC	4.37 ± 0.06aA	3.27 ± 0.29cA	2.77 ± 0.15bA
	C1	4.03 ± 0.02aB	4.87 ± 0.12aA	5.03 ± 0.47aA	4.44 ± 0.05aA	4.77 ± 0.21aA	4.10 ± 0.56aB

Data are means ±standard deviation (three independent replicates). Means followed by different letters indicate significant differences among the varieties within each storage time (lowercase letters) and among the storage times within variety (uppercase letters) (p <0.05; Tukey-Kramer’s tests).

TSS, total soluble solids; TA, titratable acidity; CA, citric acid; ni, not included in experiment. For varieties codes, see S1 Table. For ANOVA details, see S4 and S5 Tables.

For the red-ripe stage, there were no relevant changes in the color characteristics during storage in Exp#1 (Table 2). In Exp#2, significant differences were seen among the commercial variety C3, which was the most reddish (39.64 degrees), the landrace P44, which was the least reddish (72.55 degrees), and the two landraces P01 and P05 (48.34 and 46.89 degrees, respectively). A significant increase of the redness of the fruit was only seen from harvest to T10 for the two landraces P01 and P44, while the commercial variety and the landrace P05 maintained constant *H°* values across the storage times (Table 2).

The lycopene content also showed relevant variations in Exp#1, both across storage times and among varieties within storage times. Indeed, at harvest, the commercial variety C3 showed the highest lycopene content (*LYC* = 10.60 mg 100 g^-1^ FF), followed by the landraces P05 (*LYC* = 8.11 mg 100 g^-1^ FF) and P01 (*LYC* = 6.39 mg 100 g^-1^ FF). The same trend was seen at T10 and T30, with the highest lycopene content for the commercial variety C3 (*LYC* = 13.13 mg 100 g^-1^ FF) and the lowest for landrace P01 (*LYC* = 5.33 mg 100 g^-1^ FF; Table 2). The lycopene content for the red-ripe group in Exp#2 increased during storage, with no significant differences seen over time, except for landrace P01. The commercial variety (C3) again showed the highest lycopene content at each storage time (Table 2).

In general, few correlations were seen among the color parameters and lycopene contents of the fruit: for the turning stage, the lycopene content showed positive correlation with the a* values (Exp#1, Exp#2: *r* = 0.89, 0.74, respectively) and negative correlation with *H°* (Exp#1, Exp#2: r = -0.79, -0.83, respectively). This indicated that modifications to the fruit color are associated with increasing lycopene contents during the ripening process. Also, for the red-ripe stage, the a* values showed positive correlation with the lycopene content (Exp#1, Exp#2: *r* = 0.66, 0.81, respectively).

### Chemical analyses

Within the turning stage group, the pH of the tomato fruit increased, with small and inconsistent differences among the storage times for both experiments. However, landrace P46 in Exp#1 showed a significant pH increase from 4.31 at harvest to 4.45 at T20 (Table 3). In both Exp#1 and Exp#2, significant differences were seen among varieties within storage time until T20. Indeed, the commercial variety (C1) always emerged as the most acid with landraces P16 and P46 alternatively showing slightly higher pH values (Table 3).

In both years, the total soluble solids did not show considerable variations either across the storage times or among the varieties at each storage time, except for the commercial variety (C1) which showed a decrease in *TSS* during storage in Exp#1 and had the highest total soluble solids at each storage time in Exp#2 (Table 3).

The titratable acidity slightly decreased during the storage with no consistent variations except in Exp#1 for landrace P46, that experienced a significant decrease of *TA* at T20 and showed the lowest *TA* at T10, T20 and T30 (Table 3). Similarly, in Exp#2 landrace P46 consistently registered among the lowest titratable acidity values across the storage times (Table 3).

For the red-ripe stage, there were generally modest variations among the varieties and across all of the parameters (i.e., *pH*, *TSS*, *TA*, *LYC*). For pH, this was relatively stable among the varieties through both Exp#1 and Exp#2, except for the commercial variety C3 in Exp#2. This showed the highest pH at each storage time and increased pH from harvest (pH 4.34) to T30 (pH 4.55; Table 4).

**Table 4:** Chemical parameters for the red-ripe stage fruit for both experiments.

Days post-	Variety	Exp#1 (2017–18)			Exp#2 (2018–19)		
harvest	code	pH	TSS	TA	pH	TSS	TA
			(°Brix)	(g CA L^-1^ juice)		(°Brix)	(g CA L^-1^ juice)
T0	P01	4.25±0.04aA	4.00±0.10bAB	4.97±0.55aA	4.25±0.01bA	3.97±0.06cA	4.07±0.15bA
	P05	4.24±0.04aB	4.47±0.06bA	4.80±0.52aA	4.30±0.04abB	4.77±0.25bB	4.27±0.25bA
	P44	ni	ni	ni	4.29±0.02abA	4.93±0.12bA	4.23±0.31bA
	C3	4.29±0.02aA	6.53±0.32aA	4.47±0.29aA	4.34±0.02aC	7.57±0.23aA	5.70±0.20aA
T10	P01	4.27±0.05aA	4.10±0.20bAB	4.90±0.56aA	4.22±0.02bA	3.50±0.26cA	3.83±0.31aA
	P05	4.26±0.03aAB	5.23±0.92abA	4.90±0.56aA	4.25±0.04bB	4.60±0.36bB	4.03±0.68aA
	P44	ni	ni	ni	4.25±0.02bA	4.23±0.35bcA	5.27±2.14aA
	C3	4.31±0.03aA	6.57±0.25aA	4.47±0.12aA	4.38±0.03aC	7.73±0.12aA	5.27±0.15aAB
T20	P01	4.26±0.03aA	4.40±0.20bA	5.33±0.06abA	4.19±0.04bA	3.67±0.23cA	4.07±1.12aA
	P05	4.33±0.03aA	4.97±0.50bA	5.40±0.52aA	4.27±0.02bB	6.10±0.72bA	4.23±0.49aA
	P44	ni	ni	ni	4.25±0.04bA	4.07±0.15cA	4.10±0.20aA
	C3	4.34±0.03aA	6.50±0.17aA	4.57±0.12bA	4.48±0.02aB	7.70±0.20aA	4.93±0.32aBC
T30	P01	4.29±0.04aA	3.77±0.25bB	4.63±0.67aA	4.20±0.05cA	3.60±0.20bA	3.87±0.21aA
	P05	4.31±0.01aAB	5.20±1.15abA	5.63±1.02aA	4.40±0.03bA	4.37±0.45bB	3.47±0.64aA
	P44	ni	ni	ni	4.23±0.05cA	4.43±0.65bA	4.67±0.76aA
	C3	4.35±0.06aA	6.50±0.17aA	4.27±0.25aA	4.55±0.03aA	7.47±0.21aA	4.30±0.26aC

Data are means ±standard deviation (three independent replicates). Means followed by different letters indicate significant differences among the varieties within each storage time (lowercase letters) and among the storage times within variety (uppercase letters) (p <0.05; Tukey-Kramer’s tests).

TSS, total soluble solids; TA, titratable acidity; CA, citric acid; ni, not included in experiment. For varieties codes, see S1 Table. For ANOVA details, see S4 and S5 Tables.

For total soluble solids, in Exp#1 the highest values were always observed for the commercial variety C3 (mean, *TSS* = 6.5°Brix; Table 4). The commercial variety C3 showed the highest *TSS* at each storage time also in Exp#2 (mean, *TSS* = 7.6°Brix) while, among the landraces, the highest values were seen for P44 and P05, at both harvest (*TSS* = 4.9, 4.8°Brix, respectively) and T10 (*TSS* = 4.2, 4.6°Brix, respectively) (Table 4).

For titratable acidity, there were no particular changes seen for Exp#1, with landraces P01 and P05 showing the highest values (Table 4). In Exp#2, the highest titratable acidity was at harvest for the commercial variety C3 (*TA* = 5.7 g citric acid L^-1^ juice), which was significantly higher compared to the landraces (Table 4). This also showed significant variations over time for C3, as a decrease from 5.7 g citric acid L^-1^ at harvest to 4.3 g citric acid L^-1^ at T30 (Table 4).

### Weight loss

**Fig 1:**
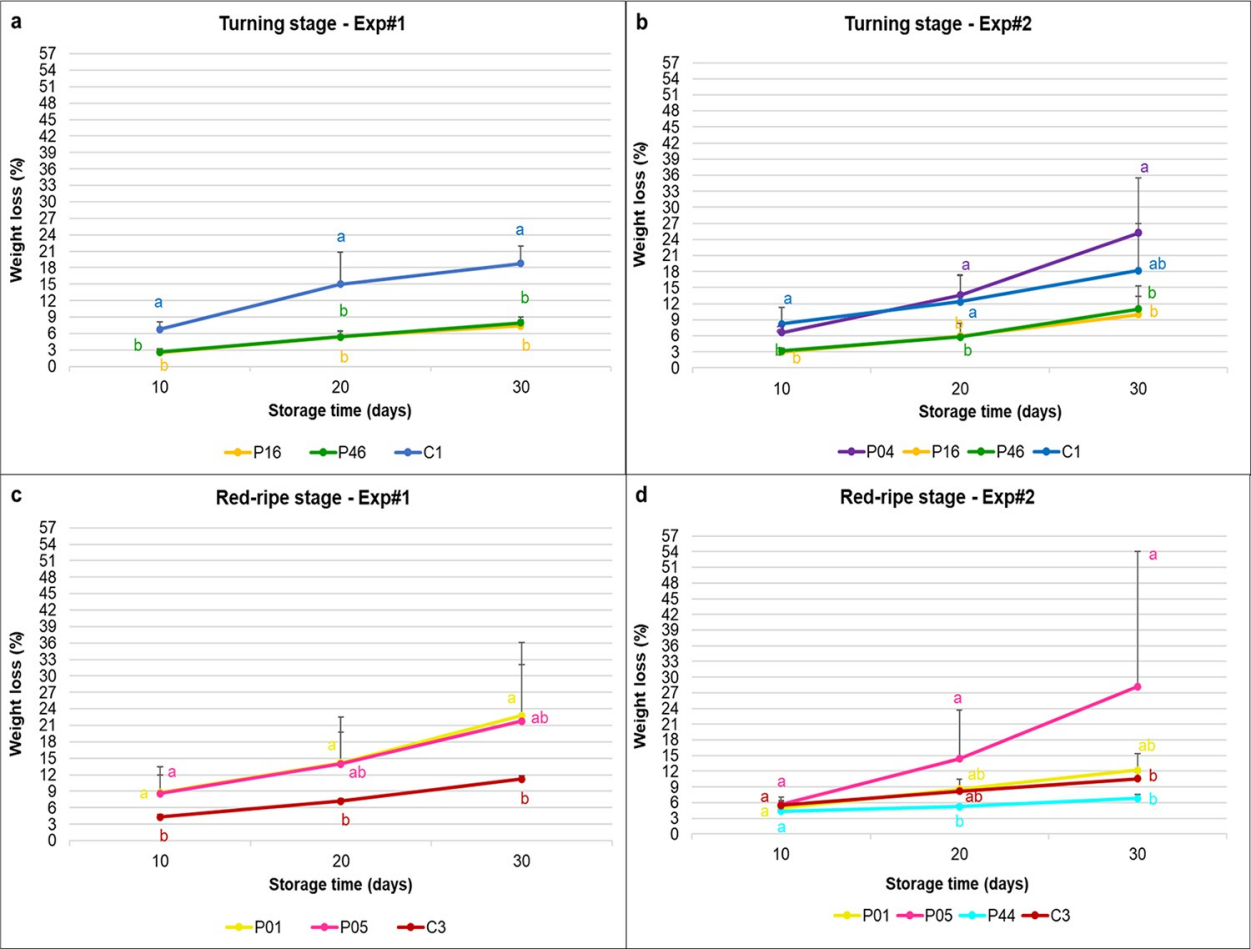
Weight loss relative to harvest weights for the turning stage fruit for Exp#1 (a) and Exp#2 (b), and for the red-ripe stage fruit for Exp#1 (c) and Exp#2 (d). Data are means ±standard deviation. Means followed by different letters indicate significant differences among the varieties within each storage time (p <0.05; Tukey-Kramer’s tests). For variety codes, see S1 Table. For ANOVA details, see S4 Table.

Weight loss increased gradually during the storage period in both stages and experiments, and the changes in the turning stage fruit were overall lower than those at the red-ripe stage (Fig 1). For the fruit harvested at the turning stage both in Exp#1 and Exp#2 (Fig 1A, 1B), the weight losses across storage times were significantly lower for the two landraces P16 and P46 than the commercial variety C1, which showed the highest weight loss in all storage times. In Exp#2, the landrace P04 showed high weight losses across storage times, comparably to the commercial variety (Fig 1B).

For the fruit harvested at the red-ripe stage in Exp#1, the two landraces P01 and P05 showed the highest weight losses at all storage times (Fig 1C). In Exp#2, while no significant differences were observed at T10 among varieties, at T20 and T30 the landrace P44 showed the significantly lowest weight loss, as it retained almost the same weight as at T10 (Fig 1D).

Overall, we observed opposite trends between the two groups: in the turning varieties, two landraces out of three (P16 and P46) showed the lowest weight loss during the storage in both Exp#1 and Exp#2 while the commercial variety C1 showed a faster decay when compared to all of the other varieties; in the red-ripe group, on the contrary, the landraces P01 and P05 lost weight more rapidly than the commercial variety C3 in both experiments and only one landrace (P44), during Exp#2, showed the lowest weight loss together with the commercial variety.

### Fruit texture

Differences were seen among these varieties in both of the groups for the parameters evaluated in the penetration tests, within storage times and within varieties among storage times (Table 5; S6 Table). For example, for the turning stage, the commercial variety C1 showed the highest forces for skin puncture (*Fp*), deformation (*Dp*) and breaking (*Wp*) at harvest, for both trials, with significant differences seen among the varieties, except for *Dp* in Exp#1. Instead, for the red-ripe stage in Exp#2, landrace P44 and the commercial variety C3 recorded the lowest *Dp* values at each storage time, as confirmed by the *Wp* values (S6 Table).

**Table 5:** Firmness (Ep) parameter for the turning stage and red-ripe stage fruit for both experiments.

Group	Variety	Firmness (*Ep*)							
	code	Exp#1 (2017–18)				Exp#2 (2018–19)			
		T0	T10	T20	T30	T0	T10	T20	T30
Turning stage	P04	ni	ni	ni	ni	1.48±0.23aA	0.52±0.10bB	0.38±0.14aB	0.29±0.06cB
	P16	1.18±0.20bA	0.78±0.26aAB	0.57±0.10aB	0.61±0.18aB	1.59±0.13aA	0.76±0.05aB	0.69±0.13aB	0.67±0.13aB
	P46	1.01±0.09bA	0.63±0.16aB	0.58±0.02aBC	0.34±0.07aC	1.37±0.17aA	0.52±0.08bB	0.59±0.19aB	0.41±0.11bcB
	C1	1.83±0.05aA	0.98±0.32aB	0.63±0.12aBC	0.43±0.02aC	1.33±0.31aA	0.99±0.12aAB	0.71±0.03aB	0.56±0.07abB
Red-ripe stage	P01	0.97±0.06bA	0.46±0.12bB	0.42±0.15bB	0.36±0.02bB	0.91±0.13bcA	0.51±0.06bB	0.41±0.03cB	0.41±0.09cB
	P05	0.82±0.12bA	0.41±0.15bB	0.34±0.12bB	0.23±0.04bB	0.80±0.06cA	0.60±0.09bB	0.38±0.11cC	0.37±0.01cC
	P44	ni	ni	ni	ni	1.64±0.12aA	1.04±0.09aB	0.65±0.06bC	0.54±0.02bC
	C3	1.25±0.07aA	1.01±0.31aAB	0.97±0.04aAB	0.77±0.14aB	1.12±0.07bA	1.17±0.02aA	0.94±0.03aB	0.7±0.02aC

Data are means ±standard deviation (three independent replicates). Means followed by different letters indicate significant differences among the varieties within each storage time (lowercase letters) and among the storage times within each variety (uppercase letters) (p <0.05; Tukey-Kramer’s tests).

ni, not included in experiment. For varieties codes, see S1 Table. For ANOVA details, see S4 and S5 Tables.

Firmness (*Ep*) of fruit significantly decreased with storage for both turning and red-ripe groups and in both experiments (Table 5). More in detail, in the turning stage during Exp#1, the commercial variety C1 showed the highest firmness (*Ep* = 1.83 N mm^-1^) at harvest, and, after that time no significant differences were found among varieties (Table 5). The data from the analysis of the firmness (*Ep*) performed for the turning stage in Exp#1 showed significantly higher values for the commercial variety C1 (*Ep* = 1.83 N mm^-1^) than for landraces P16 (*Ep* = 1.18 N mm^-1^) and P46 (*Ep* = 1.01 N mm^-1^). The *Ep* then decreased during storage, and no significant differences were seen among the varieties after T10 (Table 5). On the contrary, in Exp#2, while the varieties did not show significant differences for *Ep* at harvest, poorer performances were seen over time, with the commercial variety C1 and landrace P16 showing the highest values (*Ep* = 0.99, 0.76 N mm^-1^, respectively) (Table 5).

For the red-ripe stage in Exp#1 and Exp#2, the *Ep* was significantly different both among varieties within storage times and within varieties among storage times (Table 5). During Exp#1, the commercial variety C3 was the firmest at each storage time, with an initial *Ep* of 1.25 N mm^-1^ and a final *Ep* of 0.78 N mm^-1^ after 30 days of storage. In Exp#2, the firmest varieties were the landrace P44 and the commercial variety C3, at each storage time (Table 5).

### Visual quality

**Fig 2:**
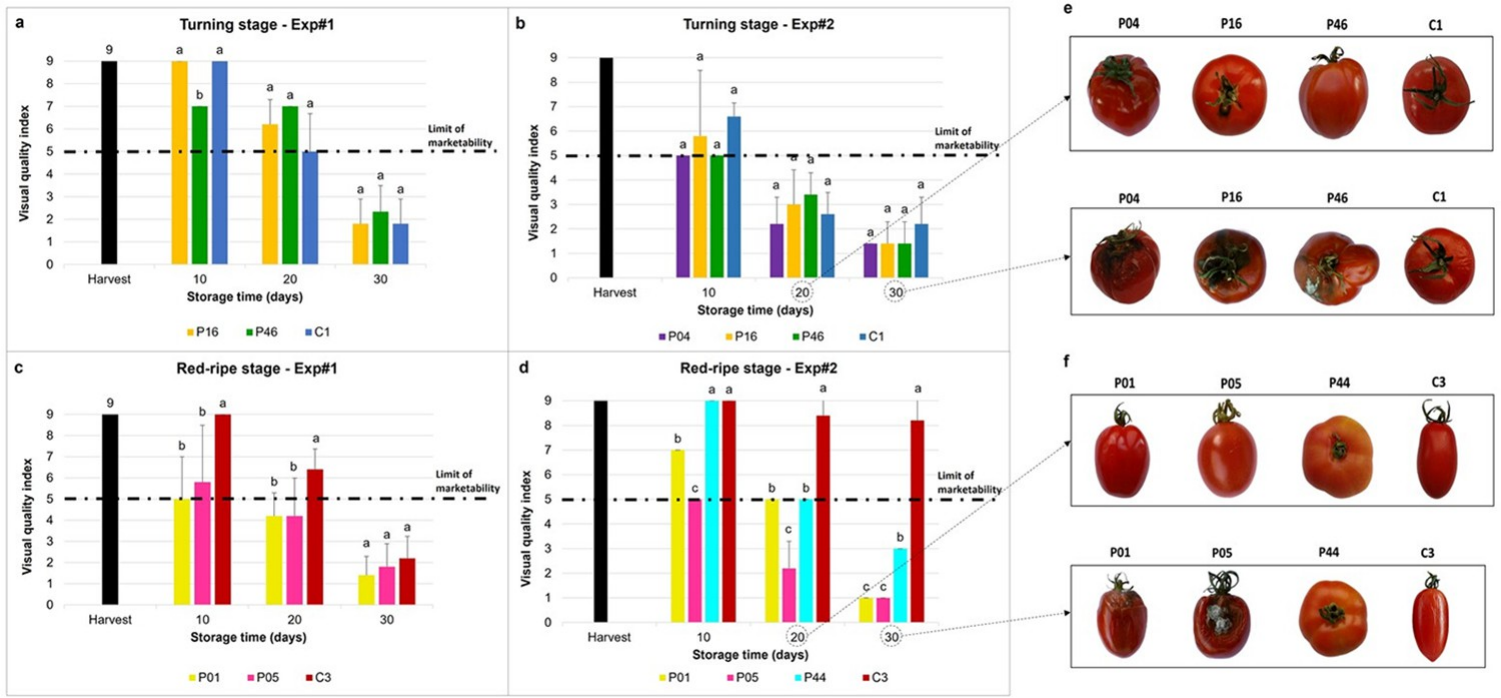
Visual quality as assessed for the turning stage fruit for Exp#1 (a) and Exp#2 (b), and for the red-ripe stage fruit for Exp#1 (c) and Exp#2 (d). On the right, examples of the changes to the tomato fruit during their shelf-life, for the turning (e) and the red-ripe stages (f) during Exp#2 after 20 and 30 days of storage. Harvest, visual quality of all of the varieties at the time of harvest (black bar; *VQ* = 9). Data are means ± standard deviation. Means followed by different letters indicate significant differences among the varieties within each storage time (p <0.05; Tukey-Kramer’s tests). When all of the fruit of a certain variety reached the same score during evaluation of the overall appearance, the standard deviations are not shown. For varieties codes, see S1 Table. For ANOVA details, see S4 Table.

The overall shelf-life according to the visual quality of the fruit varied for the two groups of Sardinian tomato landraces (i.e., turning stage; red-ripe stage) across the 2 years (Fig 2). The end of the shelf-life was declared when the mean overall appearance for each variety was below the limit of marketability (i.e., <5 points) and or when most of the fruit were rotten (Table 6, Fig 2).

**Table 6:** Shelf-life of the fruit varieties within the turning stage and red-ripe stage across both experiments.

Group	Variety	Visual quality analysis satisfied					
	code	Exp#1 (2017–18)			Exp#2 (2018–19)		
		T10	T20	T30	T10	T20	T30
Turning stage	P04	ni	ni	ni	Yes	×	×
	P16	Yes	Yes	×	Yes	×	×
	P46	Yes	Yes	×	Yes	×	×
	C1	Yes	×	×	Yes	×	×
Red-ripe stage	P01	Yes	×	×	Yes	Yes	×
	P05	Yes	×	×	Yes	×	×
	P44	ni	ni	ni	Yes	Yes	×
	C3	Yes	Yes	×	Yes	Yes	Yes

T10, T20, T30, after 10, 20, 30 days of storage, respectively; ×, no fruit satisfied visual quality analysis; ni, not included in the experiment. For varieties codes, see S1 Table.

During Exp#1, none of the varieties in either group reached 30 days of storage (Table 6). Within the turning stage fruit, only the two landraces tested (P16, P46) reached 20 days of storage, while the shelf-life of the commercial variety (C1) did not exceed 10 days due to the appearance of various pathogens on the fruit skin (Table 6, Fig 2A). In more detail, the visual quality was unchanged at T10 for landrace P16 (*VQ* = 9) and commercial variety C1 (*VQ* = 9), while it had declined a little for landrace P46 (*VQ* = 7) (Fig 2A). After 20 days, the visual quality of P16 declined (*VQ* = 6.2) to just below that of the maintained visual quality of P46 (*VQ* = 7) (Fig 2A). For the red-ripe stage, only the commercial variety C3 reached a shelf-life of 20 days (*VQ* = 6.4), while the two Sardinian landraces reached the limit of marketability at T10 (P01: *VQ* = 5.0; P05: *VQ* = 5.8) (Fig 2C).

During Exp#2, none of the varieties for the turning stage reached a shelf-life of 20 days of storage (Table 6, Fig 2B and 2E). Indeed, here the overall appearance for all of the fruit declined very rapidly, and especially for P04 and P46, which showed high proportions of fruit at the limit of marketability (*VQ* = 5) (Fig 2B) while within the red-ripe stage, the commercial variety (C3) showed the most extended shelf-life (30 days), followed by landraces P01 and P44 (20 days) and landrace P05 (10 days) (Table 6; Fig 2D and 2F). In more detail, at T10, C3 and landrace P44 showed no changes in fruit visual quality from their initial freshness, while this declined for the other two landraces (P01: *VQ* = 7; P05: *VQ* = 5). At T20, the appearance of the fruit worsened, especially for landraces P01 (*VQ* = 5) and P44 (*VQ* = 5), which were significantly different from the control (C3).

For both the turning and red-ripe stages, and in both experiments, *Ep* showed significant negative correlation with *WL* (turning stage, Exp#1, Exp#2: *r* = -0.61, -0.72, respectively; red-ripe stage, Exp#1, Exp#2: *r* = -0.83, -0.62, respectively) and positive correlation with *VQ* (turning stage, Exp#1, Exp#2: *r* = 0.69, 0.88, respectively; red-ripe stage, Exp#1, Exp#2: *r* = 0.78 for both), indicating that water loss affects fruit deterioration.

### Principal component analysis

Principal component analysis of all parameters indicated that the first two PCs cumulatively explained nearly 85% of the total variation in Exp#1 for both the turning and red-ripe stages, and 86% of the total variation in Exp#2, for both of these groups again.

**Fig 3:**
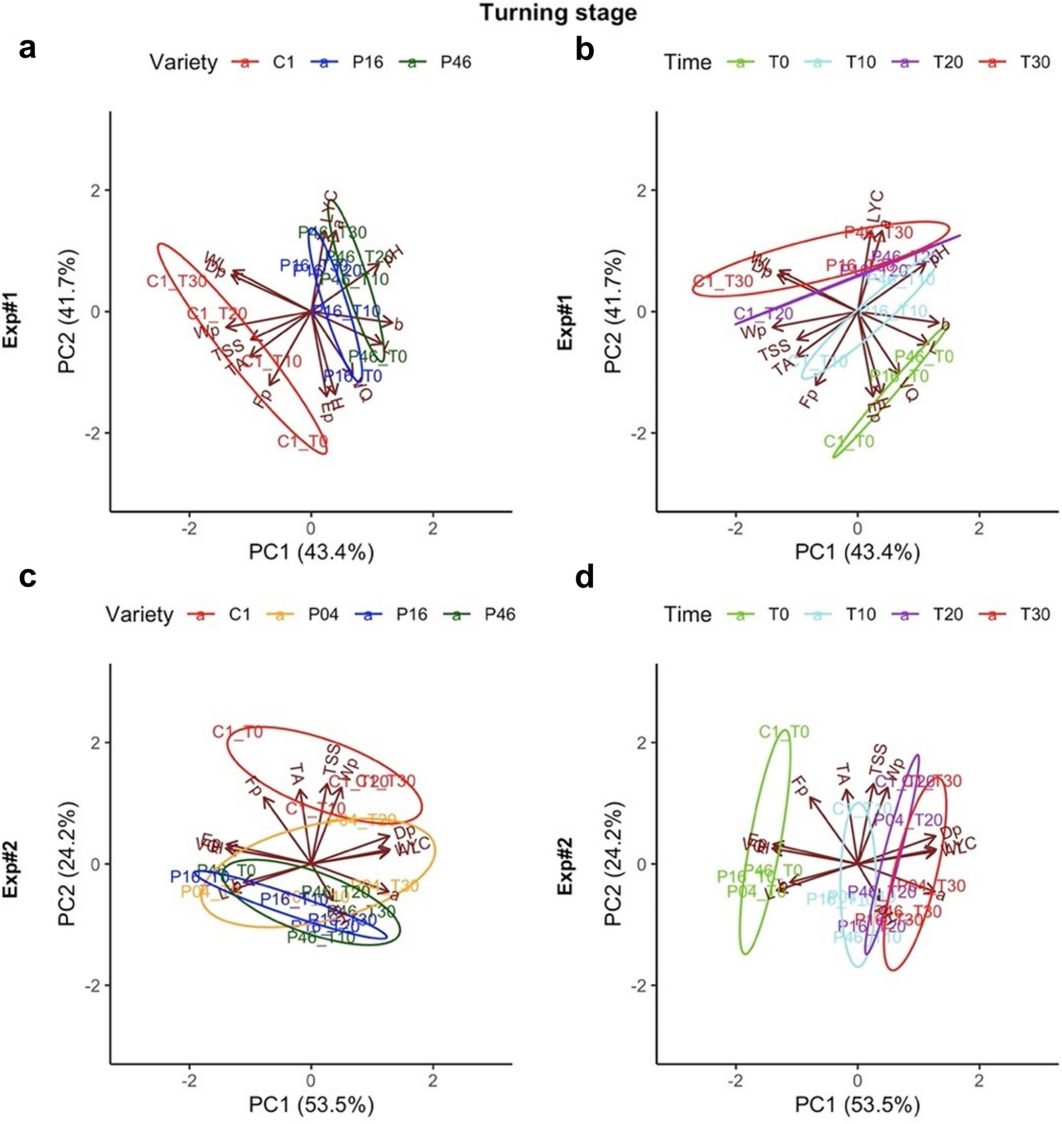
Scatter and loading (variables) plots of the first and second components obtained from the principal component analysis based on the 14 parameters within the turning stage in Exp#1 (a, b) and Exp#2 (c, d). The different colors indicate the different varieties (a, c) and storage times (b, d). T0, harvest; T10, T20, T30, after 10, 20, 30 days of storage; *WL*, weight loss [%]; *VQ*, visual quality; L* (L), lightness; a* (a), redness/ greenness; b* (b), yellowness/ blueness; *H°* (H), hue angle; *Fp*, force to puncture tomato skin [N]; *Dp*, fruit deformation before skin rupture [mm]; *Wp*, mechanical work to reach skin breaking point [N mm]; *Ep*, stiffness [N mm^-1^]; *TSS*, total soluble solids [%]; *TA*, titratable acidity [g citric acid L^-1^ juice]; *LYC*, lycopene content [mg 100 g^-1^ FF].

The first two PCs on the turning stage fruit in Exp#1 and Exp#2, highlight very similar patterns in the distributions of the varieties, also relative to the loadings of the different parameters on the PCs (Fig 3). The main difference emerged between the commercial variety C1 and landraces P16 and P46, which showed almost opposite characteristics (Fig 3A and 3C). In between these, landrace P04, which was only included in Exp#2, showed a performance more similar to landraces than to the commercial variety. The two landraces tend to group together, as they were usually characterized by the lowest values for total soluble solids (*TSS*) and titratable acidity (*TA*). In addition, they showed the lowest *Fp* and *Wp* (Fig 3A and 3C). PCA output also reveals the differentiation among the varieties across the storage times, which was particularly evident for T0, with respect to the other times. This was mainly due to a loss of weight (*WL*), visual quality (*VQ*), firmness (*Ep*), and hue angle (*H*°) during storage (Fig 3B and 3D).

**Fig 4:**
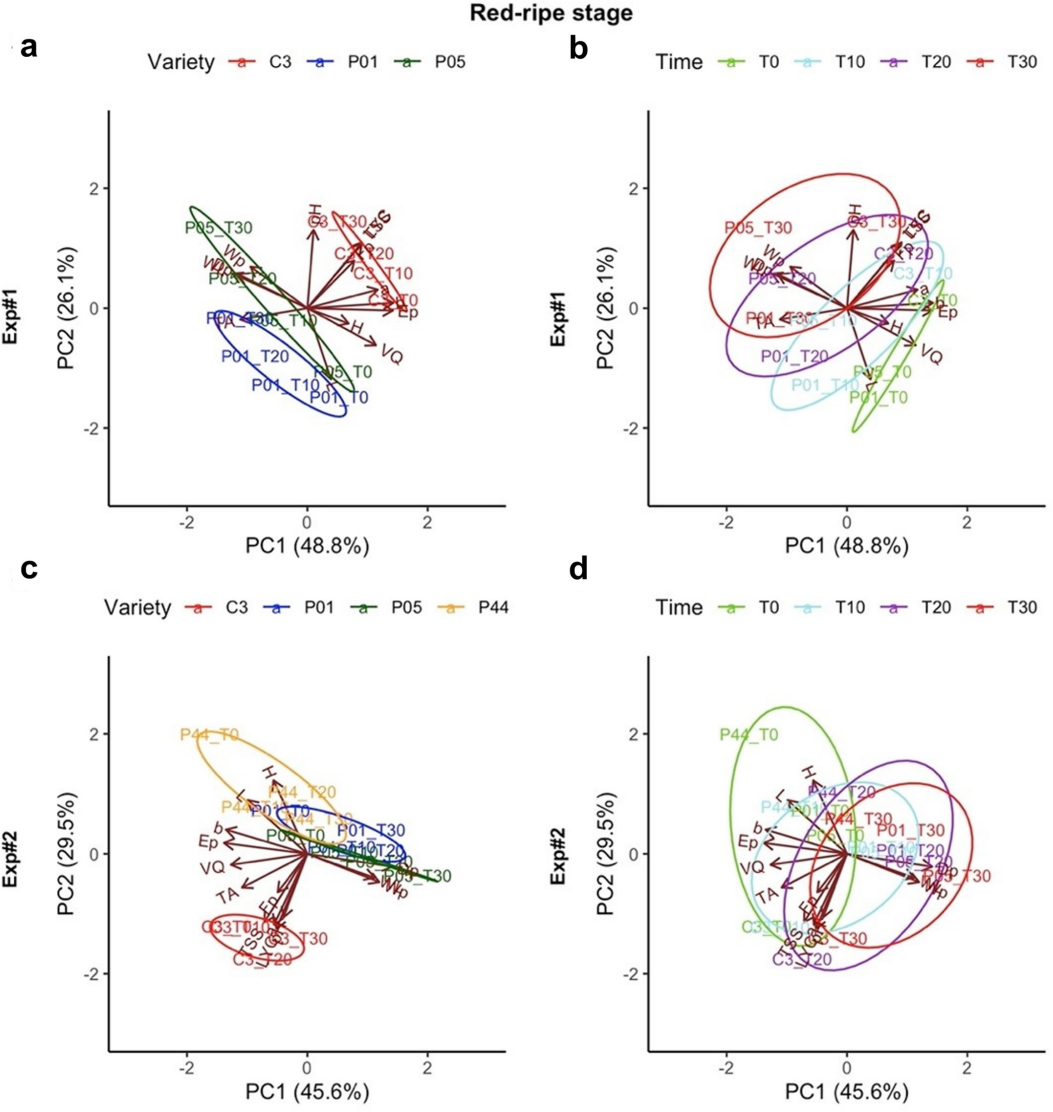
Scatter and loading (variables) plots of the first and second components obtained from the principal component analysis based on the 14 parameters within the red-ripe stage in Exp#1 (a, b) and Exp#2 (c, d). The different colors indicate the different varieties (a, c) and storage times (b, d). For abbreviations, see legend to Fig 3.

The PCA performed on the data for the red-ripe stage showed that the commercial variety C3 was clearly differentiated from the landraces for all of the parameters at each storage time, and in both experiments (Fig 4A and 4C). Landraces grouped together as they were usually characterized by the lowest total soluble solids (*TSS*), lycopene content (*LYC*), and pH during storage times (Fig 4A and 4C). Nonetheless, in Exp#2, landrace P44 emerged as being slightly different from the other landraces, especially at T0, as it was characterized by the highest L* and *H°*. When comparing the storage times similar patterns were seen for both Exp#1 and Exp#2, with no definite differentiation when passing from T0 to T30, except in Exp#1, where T0 was separated from the other times (Fig 4B and 4D).

## Discussion

The value of the different classes of crop genetic resources (crop wild relatives, landraces, and modern cultivars) has long been recognized [see e.g., 21, 69]. In tomato as for other crops, wild relatives and landraces represent a reservoir of genetic variation that can be deployed for breeding improved cultivars [70]. Modern cultivars, although less genetically diverse, are usually high-yielding and, among other important improvements, they might show for example high resistance to pests/diseases, high photosynthetic rates or extended shelf-life [2, 71, 72]. Despite this, modern cultivars usually present poor flavor and nutritional quality when compared to landraces [16, 36, 73] and there is an undoubted need to breed new varieties to answer to the new market trends and consumer’s demand.

Therefore, considering the availabilty of various tomato genetic resources, the present study focused on the different performances of landraces and commercial varieties evaluating their shelf-life in a two-year experiment. Assessing the genetic merits of landraces when fruit are harvested after their cultivation in a greenhouse autumn-winter trial might be of help for future breeding studies or for their direct valorization.

### Effects of Genotype, Year and their interaction on the Shelf-life trial

Although not many studies have evaluated the effect of the factor “Year” on tomato fruit harvested for a shelf-life experiment, sufficient results demonstrated the significant effects of genotype × environment and/or genotype x year interaction on fruit descriptors [18, 57, 74, 75]. Consistent with this, our genotype characterization at harvest revealed significant effects of Variety, Year, but also Variety by Year interaction on many of the analyzed traits. Firmness is the trait that in our study, for both turning and red-ripe stages, was not significantly affected by environment nor genotype x environment effects. The phenotypic differences might thus be directly associated to genotype, facilitating the comparison among varieties and across experiments for this trait. The present results on firmness are in line with those from Casals et al. [76] that, in a two-year trial and different environmental conditions, analyzed three long-shelf-life local varieties and two modern hybrids harvested at the red-ripe stage. These authors found that the genotype was the greatest contributor to the phenotypic variance for firmness, and that the values for this trait were significantly higher in modern varieties than in landraces [76]. Similar to the present work, more complex patterns were observed for the color and the soluble solid content, being these traits affected by significant Variety and Variety by Year interaction effects [76]. For these and the remaining traits, the performances of the varieties investigated in the present study, in terms of the responses to storage and their nutritional compositions and market-related quality attributes, have been influenced by variations in the environmental conditions that occurred in the two years in which the varieties were grown, such as light intensity, temperature, and air humidity [11, 12, 18, 54]. The significant differences seen between the two experiments for both the turning and red-ripe stages (e.g., *VQ* and *LYC* at the turning-stage) might derive from the different responses of the varieties to the different meteorological conditions that occurred over these two years. For example, during Exp#1, the total precipitation from September to December was 162 mm, while during Exp#2, this was 490 mm. During Exp#2, this might have resulted in lower temperatures and light, and in higher humidity within the greenhouse. The present outcomes thus indicate that the growing condition might significantly affect the results of a shelf-life experiment and the consequent choice of the best performing genotype, as also observed in a previous study on a local tomato landrace from Spain [74].

Most of the taste and health compounds, such as sugars and lycopene, are promoted by temperatures above 10°C during the growth period and are regulated by the spectral composition of the light [18]. Our results show that, despite the greenhouse trials being conducted during the autumn-winter season, total soluble solids and lycopene content can be considered as satisfactory for fresh tomatoes when compared to the levels usually observed under standard growing conditions (*i*.*e*., open field during the summer season; e.g., *TSS* = 4.47°Brix for mature green tomato fruit [42], compared with of *TSS* = 4.2 and 4.1°Brix for landraces P16 and P46, respectively, studied in the present study at T0 in Exp#1). Indeed, they are also similar to those seen for other environments (e.g., greenhouse during the spring/summer season) and with different management (*e*.*g*., hydroponic cultivation; *LYC* = 9.25 mg 100 g^-1^ for red maturity stage [46], compared with TSS = 8.11 and 10.60 mg 100 g^-1^ for landrace P05 and commercial variety C3, respectively, studied in the present study at T0 in Exp#1).

### Fruit color, lycopene content and chemical analyses

**Fig 5:**
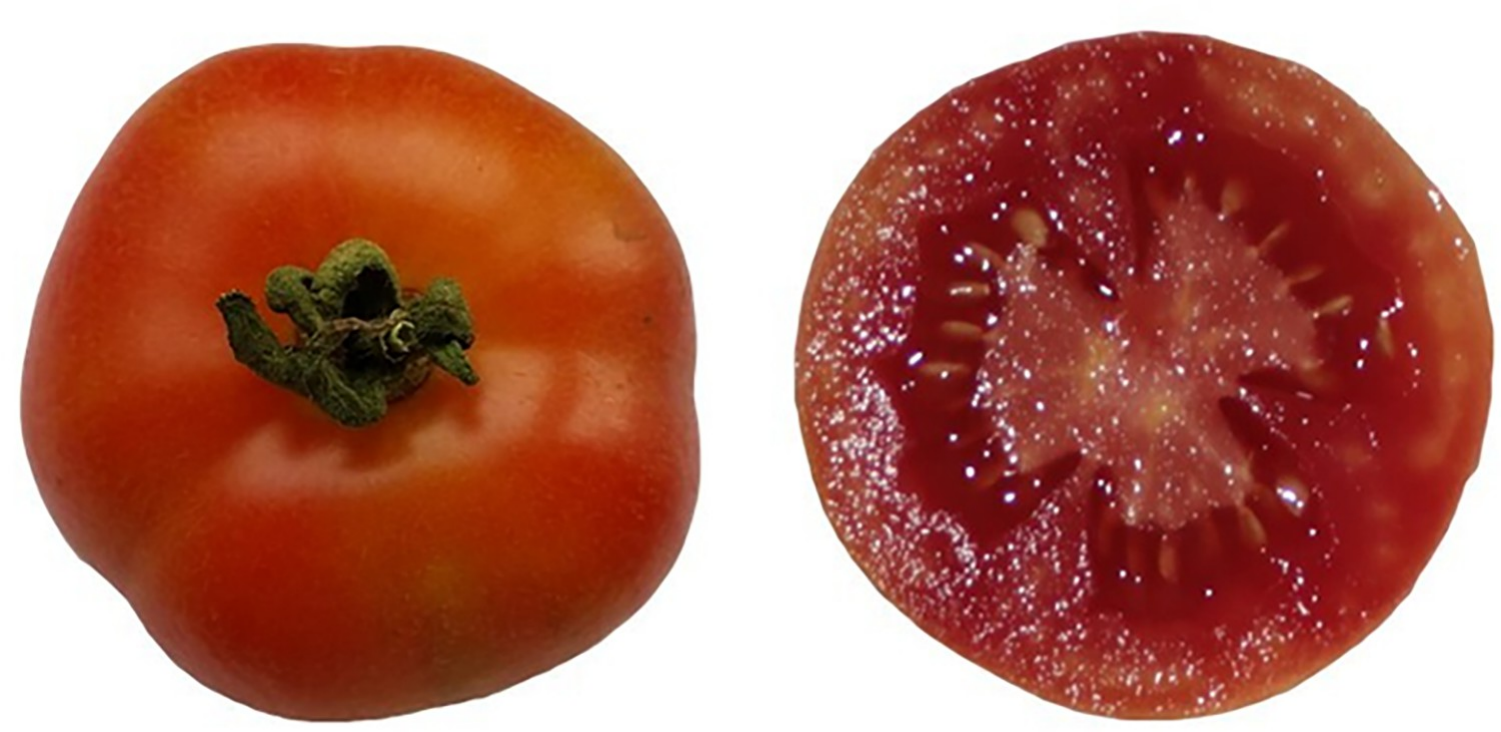
The Sardinian tomato landrace P44 that is known as ‘*Tamatta groga de appiccai*’ and is characterized at full-ripe stage by its orange skin and purple-red flesh.

Color is an important quality attribute for tomato fruit, as this greatly influences the consumer purchase, and in the present study, interesting results emerged. For example, while at the turning stage, the general decrease in the hue angle (*H°*) was expected as a consequence of the increasing maturation and as it is determined by synthesis of lycopene and degradation of chlorophyll [45, 46]. We did not expect significant variations in the color of the red-ripe group. This was true during storage in Exp#1, which is mainly because at this maturity stage the fruit are almost completely red at harvest [45]. During Exp#2, some variations were detected in the color parameters. In particular, an increase in the redness of the fruit was seen for landrace P44, which is a local variety that is characterized by an orange skin and purple-red flesh at full ripe stage (Fig 5), with this characteristic confirmed by the highest hue angle during storage (*H*° = 90°, which indicated a pure yellow color). This local variety might be an interesting product to be sell directly in the markets, because it combines an attractive color with satisfactory lycopene contents at marketable stage (6.48 mg 100 g-1 FF at T20).

An overtime increase in the lycopene content was seen for the fruit at the turning stage in both experiments, similarly to previous studies [9, 46]. On the contrary, for the red-ripe stage, the lycopene content was relatively stable during storage, with small, but not significant, increases. In addition, the few correlations seen among the color parameters and lycopene contents of the fruit indicated that modifications to the fruit color are associated with increasing lycopene contents during the ripening process. Indeed, the synthesis of lycopene occurs up to the ripe stage, with no further significant synthesis [46]. The usual lycopene content in tomatoes is ~6 mg 100 g^-1^ FF [33]. In the present study, for example, at the red-ripe stage, landraces P01 and P05 at T0 in Exp#1, and landraces P04 and P46 at T10 in both experiments, showed lycopene contents equal to or higher than this average. In our previous study [57], these landraces also showed high trans-lycopene content (10.4 and 10.6 mg/100g of fresh weight, respectively during spring/summer greenhouse cultivation). This suggests that these landraces can be used in breeding programs with the aim of developing new cultivars with high lycopene content, improving their nutritional value and the possible benefits that can be derived from its consumption [33, 77].

As well as the lycopene content, other key parameters contribute to the flavor and nutritional value of tomato, including pH, total soluble solids, and titratable acidity. Among these components, slight significant decreases in the titratable acidity were seen for landrace P46 (turning stage) and the commercial variety C3 (red-ripe stage), due to the loss of acidity and sugar content increase during the progress of fruit ripening [42, 78]. In addition, and interestingly, none of the varieties in either group showed a pH >4.4, which represents the maximum desirable limit for good flavor [32]. During the maturity process, soluble solids levels usually increase due to degradation of polysaccharides [42]. Instead, in the present study, the total soluble solids were relatively stable during the storage, with both experiments showing no significant changes for either group (turning and red ripe stage). Similar results were reported by Javanmardi and Kubota [9], where they noted modest changes in the ratio of glucose to fructose and organic acids during storage. Interestingly, among our group of landraces, the P01 showed the lowest total soluble solids, which is a characteristic that might be appreciated by people who have to control their blood insulin levels, as suggested by Renna et al. [31] for the Italian landrace ‘Regina’.

### Weight loss, fruit texture and visual quality

The significant weight loss seen during the storage for both of the groups in both of the present experiments is in line with results from previous studies; Madani et al. [79] attributed the main cause of weight loss in fruit and vegetables to increased transpiration rates and this was also observed in tomatoes stored at room temperature and controlled conditions [9, 60]. It was also found that, the weight loss in the tomato fruit increases during storage time and that individual factors and experimental conditions might influence its variation [60, 74]. This is in line with the significant differences observed between landraces and commercial varieties, because at the turning stage two landraces (P16 and P46) showed the lowest weight losses when compared to the commercial variety (C1), while the weight loss of the commercial variety (C3) and landrace P44 were among the lowest observed at the red-ripe stage. This suggests that the genotype with the best potential should be selected in each specific condition.

The texture of fleshy fruit and vegetables is another key factor that affects their marketability and, together with color, sugar, acid content and their interactions, these parameters are considered as the most important parameters that affect consumer acceptance and perception of quality [13, 56, 63].

Among the four texture parameters (*Fp*, *Dp*, *Wp*, *Ep*) that were evaluated in the present study, firmness (*Ep*) appeared to be the one that better explains the softening of the fruit. For both the turning and red-ripe stages, and also in both experiments, there were decreases in firmness during storage, which were due to increased ripeness, and also over-ripeness, of the fruit. At harvest, the varieties at the turning stage were firmer than those at the red-ripe stage, which thus confirms that the level of softening of the fruit was affected by the maturity stage at harvest time [42]. The differences that were found among varieties in both groups and experiments, especially between commercial varieties and landraces, were correlated with their weight loss and visual quality. As expected, the commercial varieties showed higher firmness than local varieties, confirming that these were improved to reduce postharvest damages [38, 76, 80]. These results are in line with previous studies that indicate that the water loss is one of the main causes of the fruit deterioration and indicate that fruit softening is related to the susceptibility of the different varieties to storage and that the decrease in firmness of the fruit might be related to the different polysaccharide degradation pathways [42, 63, 79].

As shown by the data, none of the varieties at either stage and in either Exp#1 or Exp#2 reached 30 days of storage, where most of the fruit became not marketable, except for the commercial variety C3 in Exp#2. In general, the overall appearance for all of the varieties declined over time, due to the initial emergence of defects in the fruit surface (e.g., shriveling, bruising, pitting) that gradually resulted in high proportions of rotten fruit. Based on the present results, we suggest to the researchers interested in repeating our methodology, to add an inspection time and perform the analyses at seven-day intervals from harvest time; this would likely allow to collect additional informative data on the progress of shelf-life.

### General performance of landraces and commercial varieties

The multivariate analysis, performed to simultaneously evaluate all of the quality characteristics for the different genotypes, allowed to clearly differentiate the local varieties from the commercial varieties used as the controls in both groups and experiments.

For the turning stage a distinct separation of the four storage times was evident in both years, highlighting the gradual and progressively worsening changes in the different tomato fruit quality characteristics. These changes were evident for both landraces and the commercial variety C1, but landraces were generally less acid and less sweet than the C1. In particular, landrace P16 exhibited satisfactory results in both Exp#1 and Exp#2 for weight loss, firmness and visual quality also considering that it showed lycopene levels comparable to those of the commercial variety.

In the red-ripe stage, a pronounced distance was observed between the landraces and the commercial variety C3, which was characterized by higher lycopene and soluble solid contents. The landraces tended to group together at each storage time, except for landrace P44. In particular, a significant difference between C3 and P44 is evident for the color of the fruit and the lycopene content. This landrace also showed satisfactory performances when looking at the weight loss, firmness and visual quality. On the other hand, among the evaluated landraces, the P05 was the one that showed valuable levels of lycopene and soluble solid contents. The TSS levels of our landraces measured at harvest (average 4.5%Brix), were within the range of those found in long-shelf-life landraces evaluated in protected cultivation [76], and those from three different landraces evaluated in a greenhouse trial in autumn-winter season conducted in Italy [81] but lower than those found from Figàs et al. [35] in long shelf-life landraces (average 6.3%Brix) cultivated in greenhouse during spring-summer season in Spain. The average titratable acidity of the control landraces (0.27%) found by Moles et al. [81], was 1.65 times lower than the TA levels found in our landraces (0.45% as the average of the two years), but similar to those from Figàs et al. [35]. Sugar contents, usually increase during summer and decrease during winter, while the contrary happens to the acidity of the fruit [81]. Thus, while the % Brix found in our landraces are in line with the values expected in an off-season experiment, the acidity levels were similar to those observed in other landraces grown during the spring-summer season [35].

Among the landraces investigated in the present study, P04 and P05 showed faster decay when compared to the other varieties, and especially to the commercial variety C3. These two landraces were selected overtime by local Sardinian farmers for definite traits, as suggested by their local names. Specifically, the local name of P04 is ‘*Tramatticasa tundas a siccu*’, which literally means ‘round tomato grown under low water conditions’, while the local name of P05 is ‘*lorigheddas de appiccai*’, or literally ‘rings to hang up’. Both of these landraces are mainly used as dried tomatoes after long storage under dry and dark conditions, with these tied up in circles and hung from the ceiling of a pantry. These names and uses let suppose that these landraces might be comparable to the long shelf-life landraces largely investigated in previous studies [35, 76, 82, 83]. But, in contrast to the present results on landraces P04 and P05, the fruit of the long shelf-life landraces usually remain sound for up to 6–12 months after harvest. A possible reason for this different performance might depend on the fruits storage conditions of the present shelf-life experiment (13°C and 90% relative humidity for 30 days), while long shelf-life fruit are usually harvested in the summer, fully ripen on the vine, and stored hung [82, 83]. Future shelf-life studies and quality characterization of the same local varieties might be of help to identify the best performing genotypes after their cultivation in standard conditions (in open-field or green-house during the spring-summer season). Alternatively, pre-harvest controlled conditions (such as salinity and greenhouse air CO_2_ enrichment) might be tested in an autumn-winter season to evaluate the possible effects on the quality and shelf-life of these tomato landraces in respect to commercial varieties [12, 18, 81].

## Conclusions

To the best of our knowledge, this study provides for the first time an evaluation of the responses to storage coupled to quality characterization of different tomato landraces that have been cultivated in a two-years off-season trial. Despite the growing conditions were not the standard for the cultivation of local tomato varieties, these data highlighted the potential of some of these varieties. In particular, landrace P16 exhibited satisfactory results for weight loss, firmness, visual quality and lycopene levels comparable to those of the commercial variety. Landrace P44, is an interesting product that could be sold directly in the markets, because it combines satisfactory performances when looking at the weight loss, firmness and visual quality and an attractive color with the benefits of the carotenoids.

The results on the landraces evaluated in the present study confirm the great potential for these materials and we believe that landraces still represent valuable materials, not only for future breeding studies but also to allow their direct valorization in local markets, due to their distinctive traits that can satisfy specific market needs.

## Supporting information

S1 Fig Representation of the nine-point hedonic scale used in this study to determine the overall visual quality of tomato fruit during storage for both turning and red-ripe group. 1 = very poor (severe presence of pitting, general decay [>50%] and total loss of firmness); 3 = poor (moderate presence of visual defects, 6% to 50%); 5 = good, limit of marketability (1% to 5% defective, slight loss of firmness and mild presence of shriveling); 7 = very good (no pitting and decay on the fruit surface and very slight loss of firmness); 9 = excellent (0% of damage and fruit very firm). For varieties codes, see S1 Table.
(TIF)

S2 Fig Trends of the traits for which a significant Year x Variety interaction effect was detected. Differences among the least square means (LSM) of the varieties across experiments (Exp#1 and Exp#2) were tested by using the Tukey-Kramer test. Points (i.e. LSM per each variety within experiment) marked by different letters are significantly different (p <0.05). TSS, total soluble solids; TA, titratable acidity; H°, Hue angle; LYC, lycopene; FF, fresh fruit; For varieties codes, see S1 Table.
(TIF)

S1 Table List of varieties evaluated in Exp#1 (2017–18) and Exp#2 (2018–19). L-SAR, Sardinian landrace; CV, commercial variety; ni, not included in the experiment.
(XLSX)

S2 Table List of analyses and parameters used to evaluate the shelf-life of the varieties both in Exp#1 and Exp#2. (XLSX)

S3 Table List of varieties evaluated in the greenhouse trials and the characteristics considered to include an accession in the shelf-life experimental trials. L-SAR, Sardinian landrace; VV, Vintage variety; CV, commercial variety; FRI, Flowering-ripening interval (days); FWG, Mean fruit weight (g).
(XLSX)

S4 Table One-way ANOVA to test the presence of significant differences among genotypes for each trait within each storage time. H°, Hue angle; LYC, lycopene; FF, fresh fruit; TSS, total soluble solids; TA, titratable acidity; CA, citric acid; Ep, firmness. n.s. not significant, * P < 0.05, ** P < 0.01, *** P < 0.001, **** P < 0.0001.
(XLSX)

S5 Table One-way ANOVA to test the presence of significant differences among storage time for each trait within each genotypes. H°, Hue angle; LYC, lycopene; FF, fresh fruit; TSS, total soluble solids; TA, titratable acidity; CA, citric acid; Ep, firmness. ni, not included in experiment. For varieties codes, see S1 Table. n.s. not significant, * P < 0.05, ** P < 0.01, *** P < 0.001, **** P < 0.0001.
(XLSX)

S6 Table Texture parameters for the turning and red-ripe fruit in both experiments. Data are means ±standard deviation (three independent replicates). Means followed by different letters indicate significant differences among the varieties within each storage time (lowercase letters) and among the storage times within variety (uppercase letters) (p <0.05; Tukey-Kramer’s tests). Fp, force required to puncture tomato skin (N); Dp, fruit deformation before skin rupture (mm); Wp, mechanical work necessary to reach the breaking point (N.mm). ni, not included in experiment. For varieties codes, see S1 Table.
(XLSX)

S1 Dataset All raw data of the physicochemical and texture parameters used to evaluate the shelf-life of the varieties both in Exp#1 and Exp#2 over 30 days of storage under refrigeration. WL, weight loss [%]; VQ, visual quality; L*, lightness; a*, redness/ greenness; b*, yellowness/ blueness; H°, hue angle; Fp, force to puncture tomato skin [N]; Dp, fruit deformation before skin rupture [mm]; Wp, mechanical work to reach skin breaking point [N mm]; Ep, stiffness [N mm^-1^]; TSS, total soluble solids [%]; TA, titratable acidity [g citric acid L^-1^ juice]; LYC, lycopene content [mg 100 g^-1^ FF].
(XLSX)
