# Supplementary figures and images for: Fruit quality and shelf-life of Sardinian tomato (Solanum lycopersicum L.) landraces
Source: PLoS One. 2023 Dec 8;18(12):e0290166. doi: 10.1371/journal.pone.0290166 (PMC10707699; doi:10.1371/journal.pone.0290166)

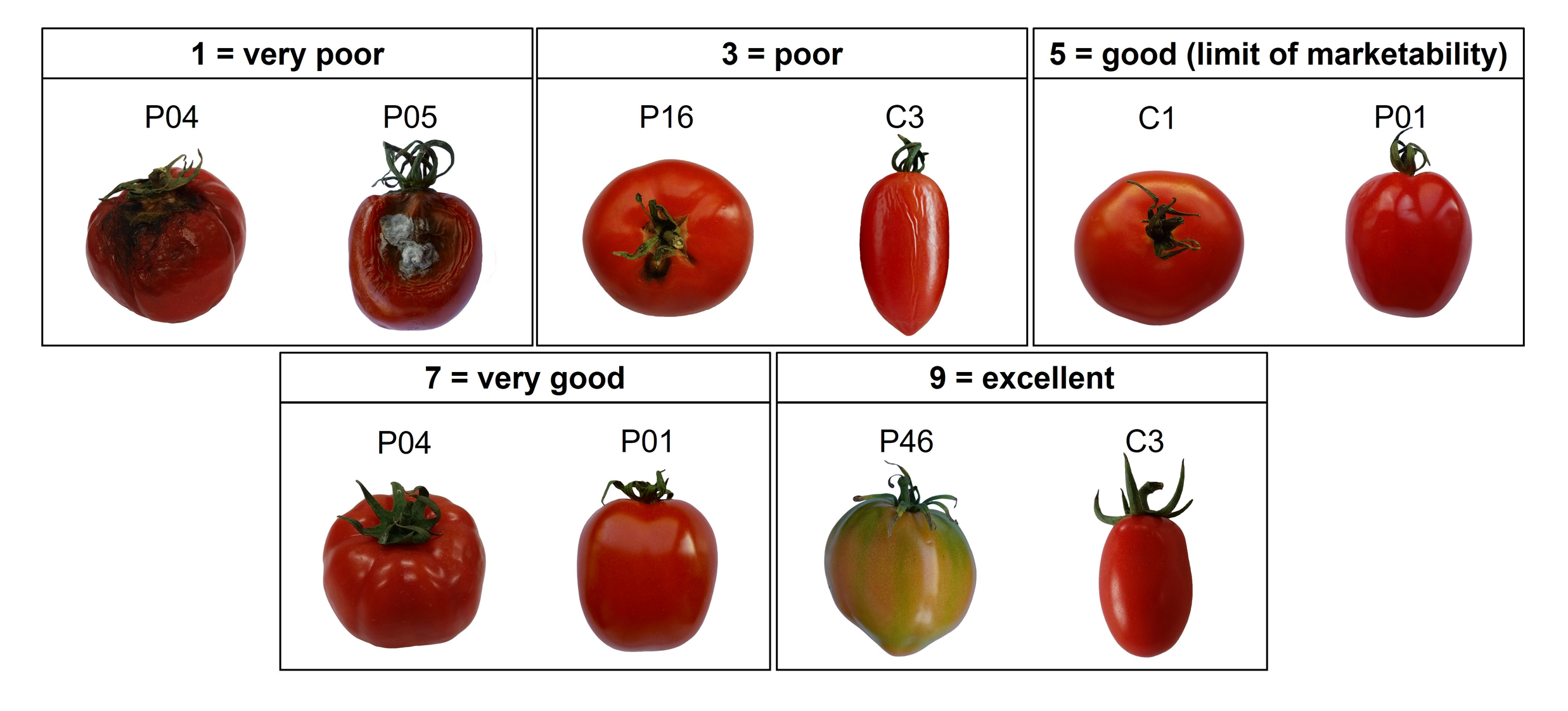

Supplement: S1 Fig — 1 = very poor (severe presence of pitting, general decay [>50%] and total loss of firmness); 3 = poor (moderate presence of visual defects, 6% to 50%); 5 = good, limit of marketability (1% to 5% defective, slight loss of firmness and mild presence of shriveling); 7 = very good (no pitting and decay on the fruit surface and very slight loss of firmness); 9 = excellent (0% of damage and fruit very firm). For varieties codes, see S1 Table. (TIF) [file pone.0290166.s001.tif]

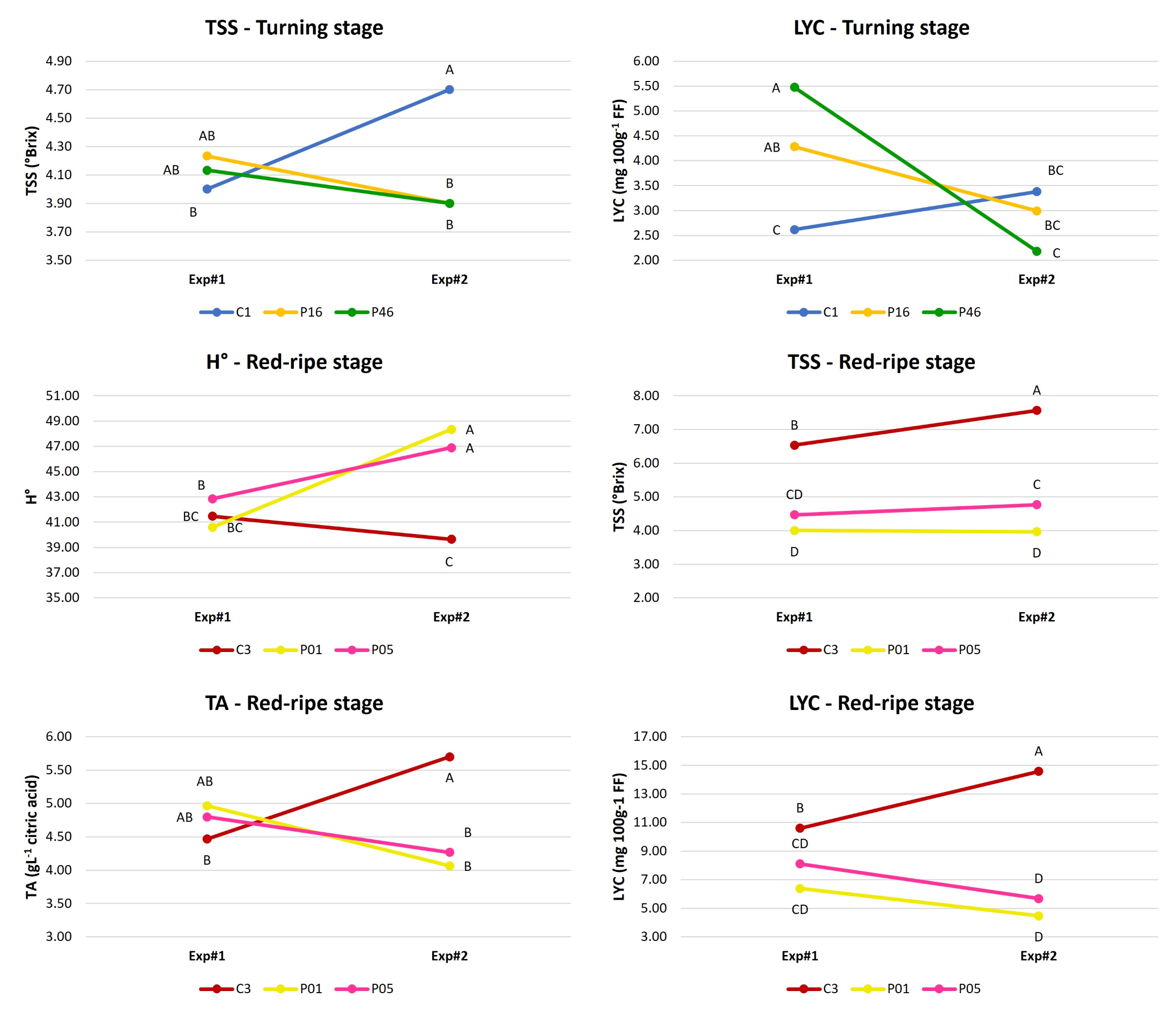

Supplement: S2 Fig — Differences among the least square means (LSM) of the varieties across experiments (Exp#1 and Exp#2) were tested by using the Tukey-Kramer test. Points (i.e. LSM per each variety within experiment) marked by different letters are significantly different (p <0.05). TSS, total soluble solids; TA, titratable acidity; H°, Hue angle; LYC, lycopene; FF, fresh fruit; For varieties codes, see S1 Table. (TIF) [file pone.0290166.s002.tif]
